# PEDOT:PSS on flexible black silicon for a hybrid solar cell on textured polyimide substrate

**DOI:** 10.1016/j.heliyon.2022.e10072

**Published:** 2022-08-02

**Authors:** Halo Dalshad Omar, Md. Roslan Hashim, Mohd Zamir Pakhuruddin

**Affiliations:** a School of Physics, Universiti Sains Malaysia, 11800 Minden, Penang, Malaysia; b Department of Physics, Faculty of Science and Health, Koya University, Koya KOY45, Kurdistan Region, Iraq; c Institute of Nano Optoelectronics Research and Technology (INOR), Universiti Sains Malaysia, 11800 USM, Penang, Malaysia

**Keywords:** PEDOT:PSS, Black silicon, Solar cells, Texturing, Polyimide

## Abstract

This work investigates properties of PEDOT:PSS on flexible black silicon (bSi) for a hybrid solar cell on textured polyimide (PI) substrate. The flexible bSi is formed by thinning down crystalline silicon (cSi) wafers to 65 μm thickness, followed by fabrication of bSi nanowires (NWs) on the wafer surface using one-step metal-catalyzed electroless etching (MCEE) technique. The resulting bSi NWs exhibit an average diameter of around 90–100 nm and length of 900 nm. Then, PEDOT:PSS with a thickness of 150 nm is coated on the flexible cSi and bSi NWs. For texturing of PI, copper-seeding technique is used. The planar and textured PI substrates are then attached to the back of the flexible cSi and bSi. The PEDOT:PSS/flexible bSi on PI substrate shows lower broadband reflection when compared to PEDOT:PSS/flexible cSi. This is due to the presence of bSi NWs on wafer surface which leads to refractive index grading effect. The PEDOT:PSS/flexible bSi solar cell on the textured PI substrate demonstrates conversion efficiency of 2.58%. This is contributed by the increased short-circuit current density (J_sc_) in the device (when compared to the device on planar PI), owing to the enhanced light absorption above wavelength of 800 nm.

## 1 Introduction

Thin and flexible black silicon (bSi) is a promising candidate to reduce silicon solar cells manufacturing costs due to its low material consumption and superior broadband light absorption within 300–1100 nm spectral region [1, 2]. The superior broadband light absorption results in a high photocurrent hence high conversion efficiency in the solar cells. The enhanced broadband absorption is attributed to presence of the random nanotextures (usually in the form of nanowires or nanopores) on the front surface of the silicon wafers with a gradually increasing refractive index which reduces broadband light reflection and improves light-coupling into the silicon absorber [3, 4, 5]. To produce flexible bSi surface, metal-catalyzed electroless etching (MCEE) technique is commonly applied to flexible silicon wafers due to its low-cost and facile fabrication process [6, 7, 8].

Conventional bSi solar cells utilize a high-temperature diffusion process (900–1000 °C) which involves a high thermal budget in order to introduce a thin diffused emitter for p-n junction formation [9]. After the high-temperature diffusion process, the nanotextures could be deformed, which leads to increased broadband reflection and therefore reduced light absorption in the spectral range of 300–1100 nm [10]. This issue can be mitigated by adopting an organic material such as poly(3, 4-ethylenedioxy-thiophene):poly(styrene-sulfonate) (PEDOT:PSS) to form the front emitter in a hybrid configuration. Besides eliminating the deformation of the nanotextures during the diffusion process, the PEDOT:PSS also reduces thermal budget due to its low process temperature requirement apart from being facile in nature [11, 12]. In [13], flexible cSi with front nanowires (NWs) was produced by MCEE. A thin layer of PEDOT:PSS mixed with Triton X-100 and dimethyl sulfoxide was then spin-coated on the NWs at 2000 rpm to form a hybrid p-n junction, followed by a 30 min annealing at 140 °C. Ultrathin and flexible solar cells with a thickness of 8.6 μm based on this architecture demonstrated short-circuit current density (J_sc_) of 18.3 mA/cm^2^, open-circuit voltage (V_oc_) of 542 mV and conversion efficiency (η) of 6.3%. Furthermore, flexible planar solar cells with the same absorber thickness exhibited J_sc_ of 15.1 mA/cm^2^, V_oc_ of 543 mV and η of 5.2%. In another study, PEDOT:PSS (containing 6 vol.% of glycerol and 0.5 wt.% of Triton X) was used as the front emitter. The PEDOT:PSS solution was spin-coated at 1800 rpm and then heated at 140 °C for 30 min to fabricate flexible PEDOT:PSS/cSi hybrid solar cells with front microscale pyramids. The flexible solar cells with the microscale pyramids exhibited η of 6.3%, while the flexible planar solar cells produced η of 4% [14]. All the above solar cells used air as back surface reflector (BSR) and they suffered from poor light absorption in the long wavelength region due to high transmission loss. This suggests that a rear supporting substrate such as polyimide (PI) with BSR capability is required to eliminate the transmission loss at the back of the solar cell (while providing mechanical support), therefore improving light absorption and efficiency of the cell. The rear substrate can be textured to enable light scattering and increase light absorption in the solar cells. To date, to our best knowledge, no work has been reported on PEDOT:PSS on flexible bSi for hybrid solar cell on textured PI substrate.

This paper investigates PEDOT:PSS/flexible bSi solar cells on textured PI substrate. Flexible cSi are produced by etching thick cSi wafers in concentrated potassium hydroxide (KOH) solution and then MCEE process is employed to fabricate bSi wafers. The surface of the PI substrate is textured by copper (Cu)-seeding method to induce light scattering and enhance light absorption in the solar cells. Surface morphological, optical and electrical properties of the PEDOT:PSS/flexible bSi on the PI substrate are then characterized. Then, the contributions of the textured PI towards absorption and J_sc_ in the solar cells are analyzed.

## 2 Experimental details

### 2.1 Fabrication of flexible bSi wafers

Mono cSi wafers (n-type, 300 μm thick, (100) orientation, resistivity 5–10 Ω cm) are used in this work. To thin down the planar cSi, the cSi wafers are dipped and etched in a 50 wt.% KOH solution at 90 °C, producing flexible planar cSi with thickness of 65 μm. The etching rate is around 80 μm/h. Subsequently, the flexible cSi wafers are cleaned by Radio Corporation of America (RCA) process to eliminate impurities (such as organic contaminants and metal oxides). Then, a thin native oxide layer is etched by immersing the wafers in a solution of 1:50 by volume of hydrofluoric acid (HF, 49%) and deionized water (DI H_2_O) for 15 s. After that, one-step MCEE technique is used to fabricate flexible bSi nanowires (NWs) by dipping the flexible wafers into a solution of silver nitrate (AgNO_3_): HF: DI H_2_O (7 ml: 9 ml: 34 ml) for 20 min at room temperature. Finally, residual silver nanoparticles (Ag NPs) are removed by dipping the flexible bSi wafers into concentrated nitric acid (HNO_3_, 60%) at room temperature for 5 min.

### 2.2 Fabrication of PEDOT:PSS/flexible bSi solar cells on textured PI substrate

**Figure 1:**
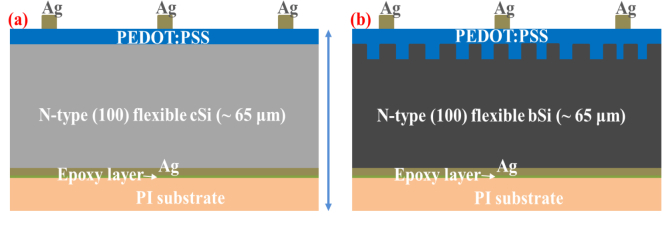
(a) Schematic diagram of the PEDOT:PSS/flexible cSi solar cell on PI substrate (b) Schematic diagram of the PEDOT:PSS/flexible bSi solar cell on PI substrate. Note that the diagrams are not to scale.

In this work, PEDOT:PSS (p-type) is deposited as the emitter on the flexible bSi (n-type) to form solar cells. Firstly, the PEDOT:PSS is mixed with 0.5 wt.% Triton X-100 and 7 wt.% ethylene glycol [15]. Then, the solution is stirred with a magnetic stirrer at room temperature for several min and the solution is filtered using a 0.22 μm syringe filter to eliminate impurities. A thin PEDOT:PSS layer is spin-coated on the flexible bSi (n-type) at 1000 rpm for 60 s, followed by annealing on a hot plate at 100 °C in air ambient for 5 min to eliminate the solvent. The thickness of the deposited PEDOT:PSS layer is around 150 nm, with a refractive index of 1.46 at 600 nm [16]. After that, Ag front and back contacts are evaporated on the solar cells by thermal evaporation. Figure 1 (a) and (b) depict a schematic diagram of PEDOT:PSS/flexible cSi solar cell and PEDOT:PSS/flexible bSi solar cell on PI substrate.

**Figure 2:**
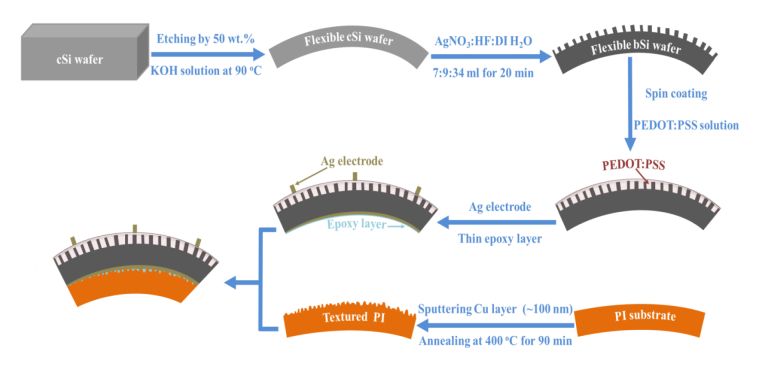
Schematic diagram for fabrication of the flexible PEDOT:PSS/bSi hybrid solar cell on textured PI substrate.

To texture PI substrate, Cu-seeding technique is used. A 100 nm Cu layer is deposited onto the planar PI substrate (with 75 μm thickness) by direct current (DC) sputtering (model: HHV, Edwards Auto 500) at room temperature, with base pressure and power of 5 × 10^−5^ mbar and 40 Watt respectively. The flow rate of incoming argon (Ar) gas is set at 10 sccm, followed by 90 min annealing at 400 °C in air environment [17]. After the annealing, the copper oxide (CuO_2_) film formed on the PI substrate is eliminated by HNO_3_ (60%) at room temperature for 20 min. Finally, both flexible cSi and bSi hybrid solar cells (with 65 μm thickness) are attached to the textured and planar PI substrates by using a thin epoxy layer. The fabrication of the flexible PEDOT:PSS/bSi hybrid solar cells on textured PI substrate is shown in Figure 2. First, cSi wafer is thinned down to 65 μm in order to produce flexible cSi. Then, flexible bSi with NWs surface are obtained by one-step MCEE process. After that, PEDOT:PSS solution is deposited as the emitter on the flexible bSi to form hybrid solar cells. The surface of the PI substrate is textured by Cu-seeding process.

### 2.3 Characterizations

The morphological properties of the PEDOT:PSS/bSi in oblique (30^o^) and cross-sectional modes are investigated by using FEI Nova NanoSEM 450 high-resolution field emission scanning electron microscopy (FESEM). The thickness of PEDOT:PSS, average diameter and length of NWs on the bSi wafers are examined by using ImageJ software. The optical properties of PEDOT:PSS/bSi on planar and textured PI substrates are evaluated using transmission and total reflection profiles in the spectral region of 300–1100 nm, as obtained by UV/Vis/NIR spectrophotometer (Cary 5000 by Agilent Technologies) with an integrating sphere. The average weighted reflection (R_w_) values of the samples are calculated using Eq. (1), where R(λ) is the total reflection and S(λ) is the AM1.5G solar spectral irradiance [18].

(1) $${\text{R}}_{\text{W}}=\frac{\int_{300\text{ ​nm}}^{1100\text{ ​nm}}\text{R}\left(\text{λ}\right)\text{S}\left(\text{λ}\right)\text{dλ}}{\int_{300\text{ ​nm}}^{1100\text{ ​nm}}\text{S}\left(\text{λ}\right)\text{dλ}}$$

Following the total reflection measurement, absorption is determined according to relationship A = (100‒R)%. Since the PEDOT:PSS/flexible bSi on PI substrate is opaque, the transmitted light through the sample can be neglected. Sheet resistance, carrier mobility and carrier concentration in PEDOT:PSS on the flexible bSi is measured by Hall effect (Model: Accent/HL 5500 PC). Current density-voltage (J-V) characteristics curve of the PEDOT:PSS/flexible bSi cells on the planar and textured PI substrates are characterized using white light-emitting diode (LED) solar simulator system (Forter Technology model TMS-2X2) under irradiance of 47 mW/cm^2^ with incident wavelengths from 400 nm to 800 nm at 25 °C. From the measurement, J_sc_, V_oc_, fill factor (FF) and η of the solar cells are determined.

## 3 Results and discussion

### 3.1 Morphological characterization

**Figure 3:**
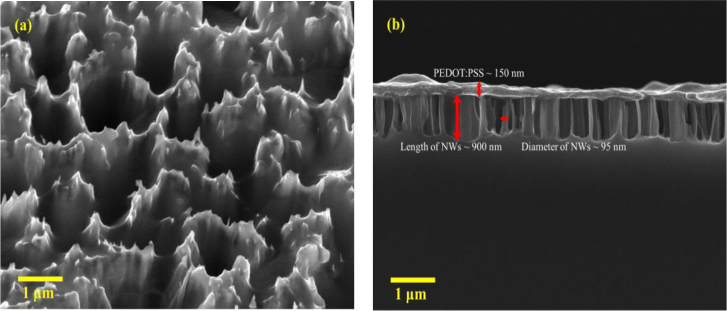
(a) Oblique (30^o^) and (b) cross-sectional view FESEM images of the PEDOT:PSS layer coated on bSi NWs.

Figure 3 depicts FESEM images of PEDOT:PSS layer on bSi NWs. In Figure 3(a), it can be observed that the PEDOT:PSS layer covers the whole surface of the bSi. Figure 3(b) presents the cross-sectional view of the PEDOT:PSS on the bSi NWs. The NWs are dense and exhibit an average length of around 900 nm and average diameter of 90–100 nm. From the cross-sectional image, it is also evident that the top PEDOT:PSS layer with a thickness of 150 nm is obtained. The PEDOT:PSS forms is uniformly distributed on top of the bSi NWs.

### 3.2 Optical characterization

**Figure 4:**
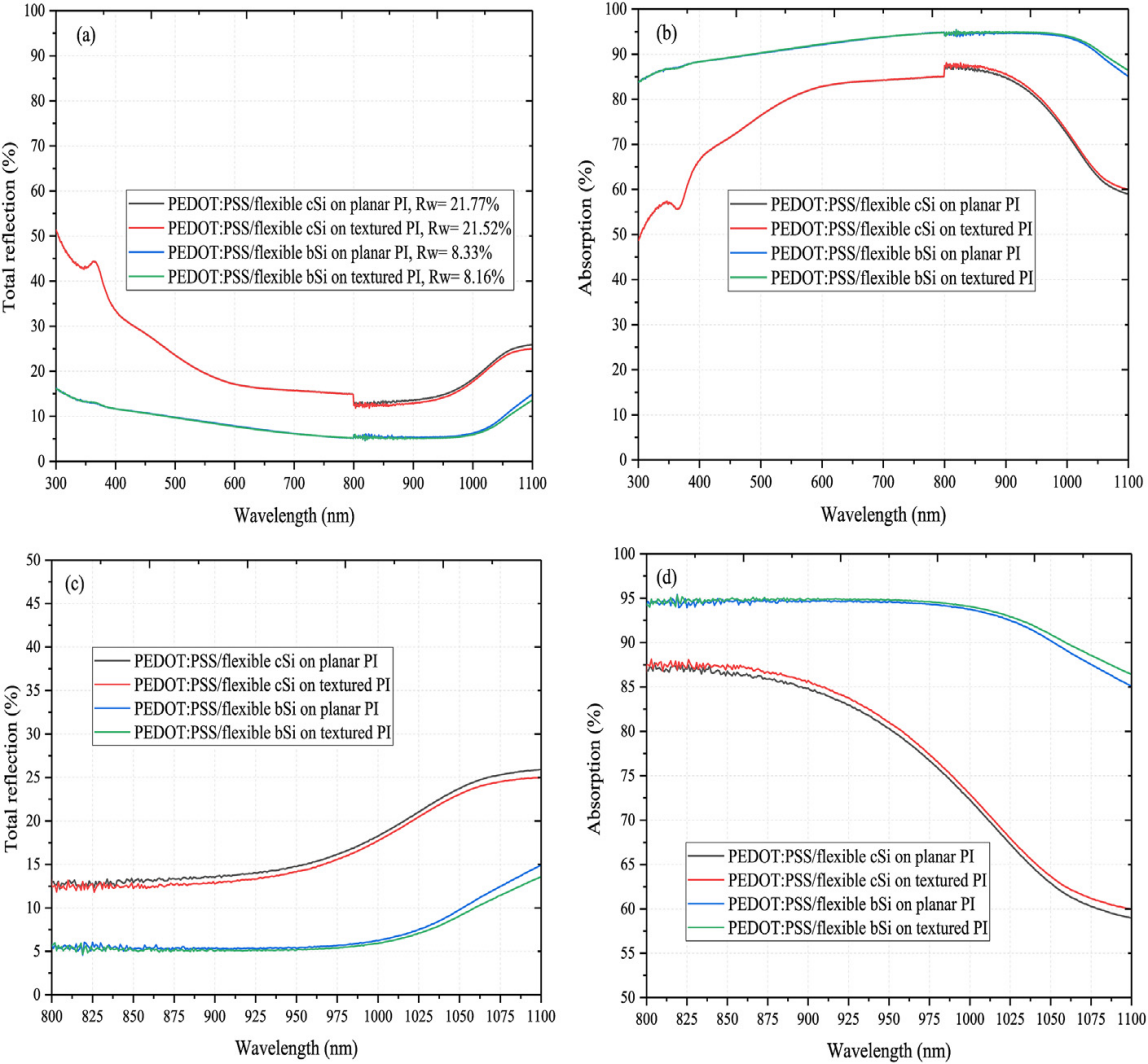
(a) Total reflection (with corresponding hemispherical R_w_ values are shown in the legend) and (b) absorption of PEDOT:PSS/flexible bSi on planar and textured PI substrates. PEDOT:PSS/flexible cSi reference on planar and textured PI substrates are shown for comparison (c) Total reflection curves for 800–1100 nm spectral region (d) Absorption curves for 800–1100 nm spectral region.

Figure 4 (a) and (b) illustrate the total reflection and absorption curves of the PEDOT:PSS/flexible bSi on planar and textured PI substrates within 300–1100 nm spectral region. The PEDOT:PSS/flexible cSi on planar and textured PI substrates are included for comparison. The R_w_ of each sample is shown in the legend. In both the total reflection and absorption curves, a small step change can be observed at wavelength of 800 nm, which is attributed to changeover of light source during the measurement [19]. The PEDOT:PSS/flexible cSi on planar and textured PI substrates illustrate reflection of about 17.14% at 600 nm due to anti-reflection effect by the PEDOT:PSS which provides an intermediate refractive index (n = 1.46) between air (n = 1.0) and the cSi (n = 4.0) [16]. The PEDOT:PSS/flexible cSi on planar PI substrate has R_w_ of 21.77% in the spectral range 300–1100 nm. With PI texturing, the reflection spectra above 900 nm is lower when compared to the planar PI substrate. This is due to enhanced light scattering by the PI textures at different angles which reduces the long wavelength reflection and leads to R_w_ of 21.52%. This results in improved absorption of around 73% at 1000 nm.

After the MCEE process, the PEDOT:PSS/flexible bSi on planar and textured PI substrates illustrates lower reflection over 300–1100 nm spectral region (with reflection of 7.7% at 600 nm wavelength) when compared to the PEDOT:PSS/flexible cSi on both planar and textured PI substrates. The reduced broadband reflection is due to the presence of the NWs on the wafer surface which reduces interfacial reflection on the front surface and improves the broadband light coupling and absorption in the wafer [6, 20]. The R_w_ for the PEDOT:PSS/flexible bSi on the planar PI is 8.33%. With textured PI substrate, the PEDOT:PSS/flexible bSi shows a slightly lower reflection and R_w_ reduces to 8.16% when compared to the PEDOT:PSS/flexible bSi on planar PI substrate, owing to enhanced scattering effect at the rear surface of the PEDOT:PSS/flexible bSi by textured PI. As a result, the light absorption increases slightly above 950 nm. The reflection and absorption curves in the long wavelength region (800–1100 nm) can be observed more clearly in Figure 4 (c) and (d) respectively.

### 3.3 Electrical characterization

Electrical properties of the PEDOT:PSS on flexible cSi and bSi are characterized by Hall effect measurement. The corresponding sheet resistance, mobility and carrier concentration values are summarized in Table 1. The PEDOT:PSS on flexible cSi demonstrates sheet resistance of 425 Ω/□ and hole concentration of 9.1 × 10^18^ cm^−3^. The hole mobility is 0.25 cm^2^/Vs. When the PEDOT:PSS is deposited on the flexible bSi NWs, the sheet resistance reduces to 398 Ω/□ and the hole concentration increases to 4.2 × 10^19^ cm^−3^. Besides, the hole mobility increases to 0.65 cm^2^/Vs. The result indicates that the PEDOT:PSS/flexible bSi cells has a lower sheet resistance than the PEDOT:PSS/flexible cSi [21]. The improvement can be attributed to the increased junction area which reveals a reasonable contact (despite not fully physical) between the bSi NWs and the PEDOT:PSS.

**Table 1:** Summary of electrical properties of PEDOT:PSS on flexible cSi and bSi as measured by Hall effect system.

Sample	Sheet resistance (Ω/□)	Mobility (cm^2^/Vs)	Carrier concentration (cm^−3^)
PEDOT:PSS/flexible cSi	425	0.25	9.1 × 10^18^
PEDOT:PSS/flexible bSi	398	0.65	4.2 × 10^19^

**Figure 5:**
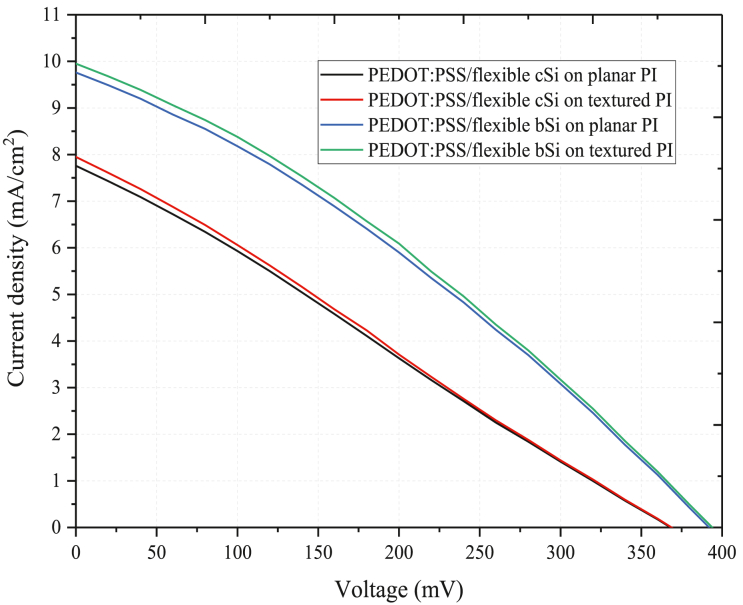
J-V curves of the PEDOT:PSS/flexible cSi and PEDOT:PSS/flexible bSi cells on both planar and textured PI substrates.

Figure 5 depicts the J-V characteristics curve of the PEDOT:PSS/flexible cSi and PEDOT:PSS/flexible bSi solar cells on both planar and textured PI substrates. The J-V parameters are summarized in Table 2. The PEDOT:PSS/flexible cSi solar cell on planar PI illustrates V_oc_ of 368.2 mV, J_sc_ of 7.76 mA/cm^2^, FF of 25.92% and η of 1.57%. For PEDOT:PSS/flexible cSi on textured PI, the J_sc_ increases by 0.2 mA/cm^2^ due to the improved long wavelength light absorption above 800 nm. η of the solar cell increases to 1.62%. For PEDOT:PSS/flexible bSi on planar PI, the J_sc_ improves by about 2 mA/cm^2^ as a result of the improved broadband light absorption and the V_oc_ increases to 392.1 mV. The efficiency of the solar cell increases to 2.51%. With the textured PI substrate, the PEDOT:PSS/flexible bSi solar cell demonstrates efficiency of 2.58% due to the increased J_sc_, owing to the enhanced light absorption above wavelength of 800 nm. Even though the J_sc_ enhancement is only 0.19 mA/cm^2^ for the bSi on the textured PI (when compared to the planar PI), the enhancement demonstrates a good correspondence with the improvement in the long wavelength light absorption (within 800–1100 nm wavelength region) for the cell on the textured PI substrate, as previously illustrated in Figure 4 (d). In our work, the efficiencies of all the solar cells are low due to relatively low J_sc_, V_oc_ and FF of the cells. The low J_sc_ can be attributed to several reasons. The J-V curves are characterized under illumination of 47 mW/cm^2^ (while standard test condition (STC) is run at 100 mW/cm^2^). It is known that the J_sc_ exhibits a proportional relationship with the level of illumination intensity [22]. So, the lower illumination intensity used in this work contributes to the lower J_sc_ in the solar cells. Besides, poor silicon sensitivity to the white LED illumination wavelength results in the low J_sc_ as well as has been observed in other works [23, 24]. The V_oc_ of the solar cells is low due to the absence of surface passivation on the surface of the solar cells [25]. The FF is low as a result of relatively resistive interface between the PEDOT:PSS and the silicon which forms the p-n junction [26].

**Table 2:** Summary from J-V characteristics of the PEDOT:PSS/flexible cSi and PEDOT:PSS/flexible bSi solar cells on both planar and textured PI substrates.

Sample	V_oc_ (mV)	J_sc_ (mA/cm^2^)	FF (%)	η (%)
PEDOT:PSS/flexible cSi on planar PI	368.2	7.76	25.92	1.57
PEDOT:PSS/flexible cSi on textured PI	368.7	7.96	25.94	1.62
PEDOT:PSS/flexible bSi on planar PI	392.1	9.76	30.85	2.51
PEDOT:PSS/flexible bSi on textured PI	393.7	9.95	31.04	2.58

## 4 Conclusions

In this work, PEDOT:PSS/flexible bSi hybrid solar cells on textured PI substrate is investigated. Concentrated KOH solution is used to thin down cSi wafers to 65 μm thickness. Then, one-step MCEE process is used to fabricate flexible bSi NWs on the wafer surface. The resulting bSi NWs demonstrate an average length of about 900 nm and average diameter of around 90–100 nm. Then, PEDOT:PSS with thickness of 150 nm is spin-coated on the bSi and cSi to form a p-n junction. To produce the textured PI substrate, Cu-seeding technique is used. The planar and textured PI substrates are then attached to the back of the planar cSi and bSi.

The PEDOT:PSS/flexible cSi on planar PI substrate demonstrates R_w_ of 21.77% within 300–1100 spectral region. After the PI texturing, the R_w_ reduces to 21.52% due to enhanced light scattering by the PI which results in the lower long wavelength reflection. After the MCEE process, the PEDOT:PSS/flexible bSi on planar PI substrate illustrates R_w_ of 8.33% as a result of lower broadband reflection, owing to NWs growth on the flexible bSi surface. On the textured PI substrate, the R_w_ reduces to 8.16%. This is contributed by the enhanced scattering effect at the textured PEDOT:PSS/flexible bSi on PI interface which reduces the reflection above 800 nm. From the Hall effect measurement, the PEDOT:PSS demonstrates lower sheet resistance, higher hole concentration and higher hole mobility on the bSi when compared to the planar cSi. The PEDOT:PSS/flexible cSi solar cell on planar PI exhibits η of 1.57%. For PEDOT:PSS/flexible cSi on textured PI, the increased J_sc_ leads to η of 1.62% in the solar cell. The PEDOT:PSS/flexible bSi solar cell on planar PI exhibits efficiency of 2.51%, due to the improved broadband light absorption. With the textured PI, PEDOT:PSS/flexible bSi solar cell demonstrates efficiency of 2.58% due to the increased J_sc_, owing to the enhanced light absorption above wavelength of 800 nm.

## Declarations

### Author contribution statement

Halo Dalshad Omar: Conceived and designed the experiments; Performed the experiments; Analyzed and interpreted the data; Wrote the paper.

Md. Roslan Hashim: Analyzed and interpreted the data; Contributed reagents, materials, analysis tools or data; Wrote the paper.

Mohd Zamir Pakhuruddin: Conceived and designed the experiments; Analyzed and interpreted the data; Contributed reagents, materials, analysis tools or data; Wrote the paper.

### Funding statement

This work was supported by 10.13039/501100004595Universiti Sains Malaysia (USM), Penang (Short-Term Grant 304/PFIZIK/6315063).

### Data availability statement

Data will be made available on request.

### Declaration of interests statement

The authors declare no conflict of interest.

### Additional information

No additional information is available for this paper.
